# Pediatric Supracondylar Humerus Fractures: Should We Avoid Surgery during After-Hours?

**DOI:** 10.3390/children9020189

**Published:** 2022-02-02

**Authors:** Sietse E. S. Terpstra, Paul T. P. W. Burgers, Huub J. L. van der Heide, Pieter Bas de Witte

**Affiliations:** 1 Department of Rheumatology, Leiden University Medical Center, 2333 ZA Leiden, The Netherlands; 2 Department of Orthopedics, University Medical Center Utrecht, Heidelberglaan 100, 3584 CX Utrecht, The Netherlands; paulepost@gmail.com; 3 Department of Orthopedics, Leiden University Medical Center, 2333 ZA Leiden, The Netherlands; h.j.l.van_der_heide@lumc.nl (H.J.L.v.d.H.); p.b.de_witte@lumc.nl (P.B.d.W.)

**Keywords:** surgery, children, orthopedics, supracondylar humerus, night, fracture, reduction, after-hours

## Abstract

Pediatric supracondylar humerus fractures occur frequently. Often, the decision has to be made whether to operate immediately, e.g., during after-hours, or to postpone until office hours. However, the effect of timing of surgery on radiological and clinical outcomes is unclear. This literature review with the PICO methodology found six relevant articles that compared the results of office-hours and after-hours surgery for pediatric supracondylar humerus fractures. The surgical outcomes of both groups in these studies were assessed. One of the articles found a significantly higher “poor fixation rate” in the after-hours group, compared with office hours. Another article found more malunions in the “night” subgroup vs. the “all groups but night” group. A third article found a higher risk of postoperative paresthesia in the “late night” subgroup vs. the “day” group. Lastly, one article reported increased consultant attendance and decreased operative time when postponing to office hours more often. No differences were reported for functional outcomes in any of the articles. Consequently, no strong risks or benefits from surgical treatment during office hours vs. after-hours were found. It appears safe to postpone surgery to office hours if circumstances are not optimal for acute surgery, and if there is no medical contraindication. However, research with a higher level-of-evidence is needed make more definite recommendations.

## 1. Introduction

Supracondylar humerus fractures account for 15% of all childhood fractures [1]. The incidence decreases sharply after the age of 10 due to skeletal maturation, and after the age of sixteen this fracture is very rare [1]. The classic trauma mechanism is a fall on the outstretched arm, resulting in an extension type fracture, which accounts for 97% of supracondylar humerus fractures [2]. A significant portion of these children need surgical treatment and fixation. However, there is debate in clinical practice whether or not to operate on these injuries during after-hours.

The main indications for acute treatment of supracondylar humerus fractures are traumatic neurovascular injury, open fractures and significant fracture dislocation [3,4]. Acute neurovascular injuries are reported in 17% of patients with a dislocated supracondylar humerus fracture [5]. Dislocation is reported in 54% and is often classified with the Gartland classification. Gartland type I indicates a fracture without dislocation, which generally can be treated conservatively. Gartland type II indicates partial dislocation, which more often requires reduction with or without fixation. Type III and IV indicate complete dislocation, where type IV also has periosteal disruption [6,7]. These type III and IV fractures are often associated with anterior interosseous nerve neuropraxia, brachial artery disruption, and other complications [1]. Both usually require closed or open reduction and fixation. Fixation is generally performed with multiple K-wire fixation [8].

Supracondylar humerus fractures are often managed at the day of admission, which can result in after-hours surgery [9]. After-hours surgery might provide additional risks for patients due to, for example, surgeon fatigue and the lack of a specialized team. In a recent publication on general orthopedic trauma, higher complication and mortality rates have been reported for surgery performed during after-hours [9]. But overall, the evidence for these alleged additional risks of after-hours surgery is limited. And in contrast, the hip fracture population has been investigated more extensively on after-hours surgery, showing no significant differences between results of office-hours and after-hours surgery [10]. However, these results cannot simply be extrapolated to the pediatric supracondylar humerus fracture patient group. Because of the lack of consensus on acute (after-hours) surgery on supracondylar humerus fractures, we investigated the following question: “Is it necessary and safe to perform surgery for pediatric supracondylar humerus fractures during after-hours?”.

## 2. Materials and Methods

We performed a literature review using the PICO methodology [11]. The following research question was applied: in children under 18 years old with a supracondylar humerus fracture (P), does after-hours surgery (I), compared with surgery during office hours (C), result in different outcomes in follow-up in terms of successful surgical treatment, function and complications (O)?

A search strategy was built in collaboration with a librarian (J. W. Schoones, Leiden University Medical Center Walaeus library). This strategy was used for PubMed, Web of Science, Embase and Cochrane to find all relevant articles written in English and published in the past 10 years (Appendix A). All references of the identified studies were evaluated for relevant articles (cross-referencing).

Studies could be included based on the following criteria: comparative study (after-hours vs. office hours) including children with supracondylar humerus fractures, reporting on clinical outcomes in follow-up, as well as radiological outcomes (successful reduction) and complications. The first selection of articles after the literature search was performed with the screening of titles and abstracts. Of the remaining articles, the full texts were evaluated for inclusion based on our eligibility criteria. Quality of evidence of the included full texts was evaluated using the GRADE criteria [12]. Additionally, articles were assessed for risk of bias in their methods and outcomes using the ROBINS-I criteria. (Appendix B) [13].

## 3. Results

The search strategy resulted in fourteen articles on November 9th, 2021. After screening of the titles and abstracts by two authors (S.E.S.T. and P.B.d.W.), nine relevant articles were identified that compared office-hours and after-hours surgeries for pediatric supracondylar humerus fractures. After reading the full texts, six of these articles could be included; in the other three, there were no reported outcomes on follow-up, succesful surgical treatment, elbow function or complications. There was no disagreement between the authors. The quality of the six articles was regarded as sufficient according to the GRADE criteria [12].

All six articles articles retrospectively compared postoperative outcomes of pediatric patients who had surgery for supracondylar humerus fractures at different times of the day. Primary outcomes in the studies were reduction quality [14,15,16,17,18,19], malunion rate [15], loss of reduction [17] and complications [16]. Also, functional outcomes in follow-up were assessed in three articles [14,15,16]. Other reported secondary outcomes were length of hospital stay, duration of surgery, rate of open surgical reduction and complications. Outcomes of the six articles are summarized in Table 1.

Aydoğmus et al. [14] compared a group of 91 children (age 0–11) diagnosed with a Gartland type III fracture without neurovascular injury in the period of January 2012 to October 2014. Of the 91 patients, 47 were operated on during office hours (8:00–17:00), and 44 during after-hours (17:00–8:00). Surgical technique was chosen by the treating surgeon. Follow-up was weekly in the first month, followed by once every three months for at least one year. 

Primary outcome was “poor fixation,” defined as pins crossing the fracture line, pins not placed bicortically, and/or pins for which the entry points were very close to each other. A significant difference in poor fixation rate was found between the groups: 4/47 patients (9%) in the office-hours group had a poor fixation, compared with 17/44 (39%) in the after-hours group (*p* = 0.005). The authors stated that a lack of sleep is often present when performing surgical treatment at night, which might lead to this higher rate. For the secondary outcomes including surgical method, placement of any medial pins, operative time, neurovascular complications, successful reduction rate, successful fixation rate, range of motion, waiting time to surgical treatment and any induced deformity, no differences were found. No reoperations were performed for any of the patients during follow-up. Potential differences in surgeon training levels and severity of the fractures between both groups were not reported. The authors concluded that surgical treatment should be performed during office hours instead of after-hours by adequately rested surgical staff. 

Paci et al. [15] included 263 patients with an uncomplicated Gartland type II, III or IV fracture diagnosed between 1 August 2002 to 31 July 2014. 263 patients with an average age of 5 years were included. Of these, 77 (29%) had surgical treatment and fixation during office hours, which was defined as 6:00–16:00 from Monday to Friday. This group was compared with 186 (71%) procedures performed during after-hours, subdivided into evening (16:00–23:00), night (23:00–6:00) and weekend (Saturdays and Sundays, 6:00–16:00). 

Primary outcome was the rate of malunion, defined as a clinically significant deformity, resulting in a change in treatment or follow-up plan. Secondary outcomes included operative time, range of motion, carrying angle and functional flexion and extension. Functional flexion was defined as ≥130 degrees, and functional extension as ≤30 degrees. A normal carrying angle was defined as 0–19 degrees of elbow valgus. On final follow-up radiographs, the Baumann angles were measured and considered normal if between 64 and 81 degrees. No significant differences were found for any of the primary and secondary outcomes after an average follow-up of 135 days. The authors reported no malunions among 77 cases in the office-hours group vs. 4 malunions of 186 (2.3%) in the after-hours group (*p* = 0.3). However, when comparing all groups to the “night” subgroup, a borderline significant difference was found for malunion: 2/236 (0.9%) in the “all groups but night” group, compared with 2/26 (9%) in the “night” group (*p* = 0.05). This outcome might be at least partially associated with the fact that there were significantly more Gartland type III/IV fractures in the after-hours group: 40/77 (73%) vs. 129/186 (57%) in the office hours group (*p* = 0.01). Furthermore, the authors found that it was more likely to have surgical treatment performed by a fellow during after-hours, compared with office hours: 72/77 (93%) vs. 95/186 (49%, *p* < 0.001). Therefore, the authors concluded that late night surgical treatment performed between 23:00 and 05:59 may be associated with a higher rate of malunion, relating it to fatigue of the surgeon, variation in training and practice patterns of the operating surgeon and experience of supporting staff. Regarding secondary outcomes, the authors reported 55/55 (100%) functional extension in the office hours group vs. 126/128 (98%) in the after-hours group (*p* = 1.00), and 68% having functional flexion in the office hours group vs. (72%) in the after-hours group (*p* = 0.6). Based on these findings, the authors caution surgeons against operating during late night hours without urgent indication. 

Wendling-Keim et al. [16]. compared 97 patients aged 0 to 18 years (mean age 5.8 years) with displaced supracondylar humerus fractures requiring osteosynthesis. Unstable Gartland type II as well as Gartland type III and IV fractures were included during a five-year period. The primary outcome was complication rate during hospital stay as well as during long-term follow-up. Complications were broadly defined, including, for example, impaired range of motion, paresthesia and wound infections. Timing of surgical treatment was recorded and stratified: daytime (7:30–16:30, 52 patients (53.6%)), early evening (16:31–22:00, 36 patients (37.1%)), late evening (22:00–2:00, 9 patients, (9.3%)) and night (2:00–7:30, no patients). The authors found that the incidence of paresthesia was significantly higher in the 22:00–2:00 group (3 out of 9 patients, 33.3%) compared with the 7:30–16:40 shift (6 out of 52 patients, 11.5%) (*p* = 0.01). No other differences in complication rates were found between office-hours and after-hours groups. Also, the authors found no association between the rate of complications and experience of the surgeon using an analysis of variance test (*p* >  0.05). It was not mentioned whether there were differences in Gartland classification between groups. 

Balakumar et al. [17] analyzed 77 pediatric supracondylar humerus fracture procedures from July 2004 to October 2009. Mean age was 7.8 years. These fractures were divided into 37 cases with surgery during office hours (8:00–20:00) and 40 during after-hours (20:00–8:00). Ten Gartland type II fractures and 67 Gartland type III fractures were included. 

The primary outcome was loss of reduction during follow-up. Secondary outcomes were number of pins used and technical quality of the pinning: i.e., adequate initial reduction, number of cortical purchases, lateral only pinning, technical errors and reduction quality (which was sufficient in case of anterior humeral line passing through the middle of the capitellum, restoration of Baumann angle and an intact medial and lateral column). Outcome evaluation was done by reviewing the intraoperative radiographs and comparing these to those acquired immediately after surgical treatment and at three weeks postoperatively. Four different pinning constructions were used, namely (a) two lateral pins; (b) three lateral pins; (c) crossed pins with one medial and one lateral entry pin; and (d) two lateral and one medial entry pin. A multivariate logistic regression analysis was performed to analyze individual factors causing loss of reduction. 

No significant difference in terms of loss of reduction was found between the office hours and the after-hours group: seven cases were found with a loss of reduction after three weeks in both the office-hours and the after-hours group, i.e., 7/37 (19%) vs. 7/40 (18%, *p* = 1.00). The article did not report any differences in secondary outcomes between both groups. Assessing the patient group with loss of reduction, lateral pinning (odds ratio: 7.73, *p* = 0.029) and technical errors (odds ratio: 57.63, *p* = 0.001) were associated with loss of reduction. No associations were found with number of pins used, adequate initial surgical treatment, number of cortical purchases and technical quality of pinning. The Gartland classifications and surgeon level of experience were not reported separately for the office-hours and after-hours group. 

The authors suggest that loss of reduction following fracture fixation is closely related to technical errors, which often results in inadequate reduction. However, as these technical errors were evenly distributed between office hours and after-hours, the authors concluded that timing of the procedure was not associated with loss of reduction.

Okkaoglu et al. [18] investigated 150 Gartland type III fracture surgeries (mean age 5.9 years (Standard Deviation (SD) 2.6). Open fractures, fractures associated with vascular injury and compartment syndrome and flexion type fractures were excluded. Of all patients, 79 underwent surgical reduction during office hours (8:00–17:00) and 71 during after-hours (of which 51 during the evening (17:00–24:00) and 20 during the night (24:00–8:00)). The office-hours surgery group partially consisted of fractures admitted during office hours, and partially of fractures postponed from after-hours. All surgeries were performed by an experienced orthopedic surgeon within 24 h of admission. The main outcome was reduction quality on postoperative radiographs, which was assessed with the lateral capitellohumeral angle (defined as acceptable: 22–70 degrees), Baumann angle (56–86 degrees) and anterior humeral line (crossing mid-third of capitellum). Other outcomes were operative time and open reduction rate.

No significant differences in patient characteristics were found between the office-hours and after-hours groups, and no significant differences were found for reduction quality, open reduction rate and mean operative time between office-hour and after-hours surgeries. Therefore, surgical outcomes were regarded as comparable between the office-hours and after-hours groups by the authors. Time from admission to surgery, however, was significantly longer for the office-hours group (14.0 (SD 5.1) vs. 6.0 (3.5) hours (*p* < 0.01)). No information on long term follow-up or functional outcomes was reported.

Based on this information, the authors concluded that it is generally safe to postpone surgery to office hours if there is no indication for acute surgery (open fractures, neurovascular impairment, compartment syndrome). However, the authors caution against very long time from admission to surgery (>24 h) as their data does not provide information on this topic.

Tuomilehto et al. [19] assessed 200 fractures (mean age 7.1 years, range 1.8 to 14.1, 15.5% Gartland type II, 83.5% Gartland type III). Of these fractures, timing of surgery for the first 100 patients depended on circumstances, and could therefore be during the night (24:00–7:00) (12% of surgeries). For the next 100 patients, a protocol was implemented postponing night-time surgery to office hours. During this period, only fractures with compromised circulation were treated during after-hours (2%). Main outcomes were pin fixation quality, number of open reductions, radiographic alignment, and postoperative complications. Pin fixation quality was regarded sufficient if both K-wires punctured both bone fragments and if pins were not crossing at the fracture line. Radiographic alignment was regarded sufficient if the Baumann angle was within ±10° of reported normal range, and the anterior humeral line crossed the capitellum with no signs of malrotation.

The authors found no significant differences between the two treatment groups before vs. after implementation, respectively: adequate pin fixation quality (42% vs. 55% (*p* = 0.08), Gartland classification (79% Gartland III vs. 88%, *p* = 0.13), Bauman angle (55% normal vs. 70% normal, *p* = 0.07)), nerve injury (14 vs. 21, *p* = 0.26), other complications (5 vs. 3 infections (*p* = 0.72), and other outcomes. No information on long term follow-up or functional outcomes was reported. It is important to note that in the postponed group, the consultant was more often the primary surgeon (43% vs. 27% (*p* = 0.02)). Additionally, in the postponed group, mean operative time decreased by 11 min (no statistics reported) and the percentage of operations shorter than 60 min decreased from 67% to 84% (*p* = 0.01).

The authors concluded that postponing supracondylar humerus surgeries to office hours may not make a difference for the patients if surgery is performed within the first 24 h, while being beneficial in terms of consultant attendance and operative time.

## 4. Discussion

Obtaining immediate reduction and supposedly optimal clinical outcome are often arguments to perform acute surgical treatment, even during after-hours, for pediatric supracondylar humerus fractures. However, most surgeons prefer to postpone surgery to office hours, as it is generally assumed that after-hours surgery provides additional risks for patients [20]. We found six relevant retrospective articles reporting on outcomes of office-hours and after-hours surgery in pediatric patients with supracondylar fractures.

### 4.1. Primary Outcomes

Aydogmus et al. [14] found a significantly higher “poor fixation” rate in the after-hours group, compared with the office-hours group. However, this higher rate of “poor fixation” was not associated with loss of range of motion in the after-hours group during follow-up. Paci et al. [15] demonstrated a borderline significant difference in malunion rates when comparing their “all groups but night” subgroups with the “night” (23:00–6:00) subgroup, with the latter demonstrating a higher malunion rate. However, there was a significantly higher Gartland classification in the concerning night group, compared with the other groups. Therefore, more severe fractures appear to have been treated with minimal delay, i.e., during after-hours, if deemed necessary by the surgeon (confounding by indication). In addition, more surgeries were performed by a fellow at night (performance bias). Both are potential confounders for the identified inferior outcomes of the “night” group. In the article of Wendling-Keim [16], surgical treatment from 22:00–2:00, compared with 7:30–16:40 was associated with a higher rate of paresthesia. The studies of Balakumar et al. [17], Okkaoglu et al. [18] and Tuomilehto et al. [19] found no significant differences between office hours and after-hours for reduction quality, loss of reduction and pin fixation quality.

### 4.2. Secondary Outcomes

No clinically or statistically significant differences were described between office and after-hours groups with regards to range of motion, carrying angle, surgical method, functional flexion and extension and any induced deformity. However, the study of Tuomilehto did show a decrease in operative time in the postponed/office-hours group (11 min), which coincided with the consultant orthopedic surgeon being more often the primary surgeon in this group (43% vs. 27% (*p* = 0.02)).

### 4.3. Early vs. Delayed Surgical Treatment

The aforementioned results suggest that the influence of timing of surgery on radiological and functional outcomes in pediatric supracondylar humerus fractures is limited. Still, in order to draw more definite conclusions about this topic, it is also important to assess differences in outcomes between direct surgical treatment and delayed surgical treatment, regardless of day and night. This is due to the fact that postponing surgical treatment to office hours implies a delay in surgical treatment. However, in our literature evaluation the articles of Aydogmus et al. [14], Wendling-Keim et al. [16] and Okkaoglu [18] assessed waiting time, but reported no association between waiting time and inferior radiological or clinical outcomes. Furthermore, an extensive review was published on delaying supracondylar humerus fracture surgery, regardless of day and night [21]. This review assessed the outcomes of 1735 patients from 12 articles, and evaluated the functional outcomes of early surgical treatment compared with delayed surgical treatment. The findings of this review are in accordance with our results, as the authors found no strong evidence that delaying surgical treatment influences the outcomes of surgery negatively or positively. This was confirmed by a more recent article of Shon et al. on Gartland type III fractures, in which no differences were reported for clinical outcomes of early and delayed surgery, as long as performed within 24 h [22].

### 4.4. Strengths and Limitations of This Study

To our knowledge, this is the first review that compares the outcomes of surgical treatment during office hours vs. after hours for pediatric supracondylar humerus fractures. There are some factors that should be taken into consideration when interpreting our results. As for all review studies, our study is as strong as its included literature. First of all, only six relevant articles were found. All of these are retrospective and with methodological limitations. Also, four out of six articles have a serious risk of bias according to the ROBINS-I criteria. Furthermore, the aforementioned confounding by indication is likely to be present. Paci et al. [15] reported this effect and was the only study making an effort to minimize this bias by using multivariable logistic regression in order to control for differences in baseline characteristics. The aforementioned performance bias is also likely to be present in the included articles. The studies of Aydogmus et al. [14] and Tuomilehto et al. [19] indeed found more surgeries performed by a fellow during after-hours compared with office hours. Wendling-Keim et al. [16] reported no significant difference, however, in the rate of complications between more and less experienced surgeons. The other studies did not report on this topic. Other limitations to the current study are that all six studies have relatively small patient groups, and quite heterogeneous outcome measures.

### 4.5. Conclusions and Recommendations

We found weak evidence for inferior reduction quality and more complications when performing after-hours surgery, which might be caused by confounding by indication. Clinical implications of this weak evidence seem limited: no significant differences were reported in any of the articles for other outcomes in follow-up, including complications and postoperative elbow function. Postponing surgery to office-hours generally coincides with the consultant surgeon more often as the primary surgeon, and with shorter operative time. Additionally, literature comparing acute vs. delayed surgery of pediatric supracondylar humerus fractures reveals no significant influence on surgical outcome.

In conclusion, it appears generally safe to postpone surgery for Gartland type II, III and IV fractures to office hours if circumstances are not optimal for surgery (e.g., no dedicated surgeon available), and if there is no medical contraindication. However, as literature on this topic is scarce and subject to various biases, more research with a higher level-of-evidence is needed to make more definite conclusions and recommendations for clinical practice. Long-term results, functional scores and the influence of surgeon experience would be key topics for further research on supracondylar humerus fractures.

## Figures and Tables

**Table 1:** Outcomes and results of the included articles.

	Patients in Office-Hours Group	Patients in After-Hours Group	Primary Outcome *	Other outcomes	Primary Result	Secondary Results	Risk of Bias According to the ROBINS-I Criteria [13]
Aydogmus et al. [14] 2017	47	44	poor fixation	surgical method, placement of any medial pins, operative time, any postoperative neurovascular complication, successful reduction rate, successful fixation rate, any induced deformity and rate of loss of function	significantly poorer fixation in the after-hours group vs. office hours. (4/47 (9%) vs. 17/44 (39%) (*p* = 0.005))	no significant differences between groups	serious risk
Paci et al. [15] 2018	77	186	malunion	surgeon subspecialty, operative time, range of motion, carrying angle and other clinical outcomes.	more malunion in the “night” subgroup vs. the “all groups but night” group (2/26 (8%) vs. 2/236, (1%, *p* = 0.05))	more surgeries performed by a fellow during after-hours, compared with office hours: 72/77 (93%) vs. 95/186 (49%, *p* < 0.001), more Gartland type III/IV fractures in the after-hours group compared with office hours: 40/77 (73%) versus 129/186 (57%, *p* = 0.01).	moderate risk
Wendling-Keim et al. [16] 2019	52	47	Complications	-	significantly more paresthesia in the 22:00–2:00 group (3/9, 33.3%) vs. the 7:30 –16:40 group (6/52, 11.5%) (*p* = 0.01)	-	serious risk
Balakumar et al. [17] 2012	37	40	loss of reduction	number of pins used and technical quality of pinning	no significant difference in loss of reduction in the office hours vs. after-hours group (7/37, (19%) vs. 7/40, (18%) *p* = 1.00)	no significant differences between groups	serious risk
Okkaoglu et al. [18] 2021	79	71	reduction quality	operative time, open reduction rate and time to surgery	no significant differences in any measures of reduction quality (*p* > 0.05)	More time to surgery during office hours; 14.0 h (SD 35.2) vs. 6.0 h (SD 3.5, *p* < 0.001)	moderate risk
Tuomilehto et al. [19] 2018	100	100	pin fixation quality	number of complications, number of open reductions and operative time	no significant difference in sufficient pin fixation quality for office hours versus after-hours (42% vs. 55% (*p* = 0.08))	operative time <60 min: 67% vs. 84% after implementation postponement protocol (*p* = 0.01)	serious risk

* not all articles explicitly mentioned their primary and secondary outcomes. In this case, the most relevant outcome to our topic was assessed as primary outcome. SD = Standard Deviation, h = hours, min = minutes.

## Data Availability

Not applicable.

## Appendix A

*Pubmed Search strategy of the present study (conducted April 2021)*

((“supracondylar humerus fracture”[tw] OR “supracondylar humerus fractures”[tw] OR “supracondylar humeral fracture”[tw] OR “supracondylar humeral fractures”[tw] OR ((“Humeral Fractures”[Mesh] OR “humerus fracture”[tw] OR “humerus fractures”[tw] OR “humeral fracture”[tw] OR “humeral fractures”[tw]) AND (“supracondylar”[tw] OR supracondyl*[tw])) OR supracondyl*[tw]) AND (“Night Care”[mesh] OR “night”[tw] OR “nights”[tw] OR “nighttime”[tw] OR “night time”[tw] OR night*[tw] OR “overtime”[tw] OR “after-hours”[tw] OR “Sleep Deprivation”[mesh] OR “Shift Work Schedule”[mesh] OR “office hours”[tw] OR “daytime”[tw] OR nocturnal*[tw]) AND (“child”[tw] OR “Child”[Mesh] OR “child”[tw] OR “children”[tw] OR “Infant”[Mesh] OR “infant”[tw] OR “infants”[tw] OR “infancy”[tw] OR “newborn”[tw] OR “newborns”[tw] OR “new-born”[tw] OR “new-borns”[tw] OR “neonate”[tw] OR “neonates”[tw] OR “neonatal”[tw] OR “neo-nate”[tw] OR “neo-nates”[tw] OR “neo-natal”[tw] OR “neonatology”[tw] OR “NICU”[tw] OR “premature”[tw] OR “prematures”[tw] OR “pre-mature”[tw] OR “pre-matures”[tw] OR “preterm”[tw] OR “pre-term”[tw] OR “postnatal”[tw] OR “post-natal”[tw] OR “baby”[tw] OR “babies”[tw] OR “suckling”[tw] OR “sucklings”[tw] OR “toddler”[tw] OR “toddlers”[tw] OR “childhood”[tw] OR “schoolchild”[tw] OR “schoolchildren”[tw] OR “childcare”[tw] OR “child-care”[tw] OR “young”[tw] OR “youngster”[tw] OR “youngsters”[tw] OR “preschool”[tw] OR “pre-school”[tw] OR “kid”[tw] OR “kids”[tw] OR “boy”[tw] OR “boys”[tw] OR “girl”[tw] OR “girls”[tw] OR “Adolescent”[Mesh] OR “adolescent”[tw] OR “adolescents”[tw] OR “adolescence”[tw] OR “pre-adolescent”[tw] OR “pre-adolescents”[tw] OR “pre-adolescence”[tw] OR “schoolage”[tw] OR “schoolboy”[tw] OR “schoolboys”[tw] OR “schoolgirl”[tw] OR “schoolgirls”[tw] OR “pre-puber”[tw] OR “pre-pubers”[tw] OR “pre-puberty”[tw] OR “prepuber”[tw] OR “prepubers”[tw] OR “prepuberty”[tw] OR “puber”[tw] OR “pubers”[tw] OR “puberty”[tw] OR “puberal”[tw] OR “teenager”[tw] OR “teenagers”[tw] OR “teens”[tw] OR “youth”[tw] OR “youths”[tw] OR “underaged”[tw] OR “under-aged”[tw] OR “Pediatrics”[Mesh] OR “Pediatric”[tw] OR “Pediatrics”[tw] OR “Paediatric”[tw] OR “Paediatrics”[tw] OR “PICU”[tw] OR (“child”[all fields] NOT child[au]) OR children*[all fields] OR schoolchild*[all fields] OR “infant”[all fields] OR “infants”[all fields] OR “infancy”[all fields] OR adolesc*[all fields] OR pediat*[all fields] OR paediat*[all fields] OR neonat*[all fields] OR toddler*[all fields] OR “teen”[all fields] OR “teens”[all fields] OR teenager*[all fields] OR preteen*[all fields] OR newborn*[all fields] OR postneonat*[all fields] OR postnatal*[all fields] OR “puberty”[all fields] OR preschool*[all fields] OR suckling*[all fields] OR “juvenile”[all fields] OR “new born”[all fields] OR “new borns”[all fields] OR new-born*[all fields] OR neo-nat*[all fields] OR neonat*[all fields] OR perinat*[all fields] OR underag*[all fields] OR “under age”[all fields] OR “under aged”[all fields] OR youth*[all fields] OR pubescen*[all fields] OR prepubescen*[all fields] OR “prepuberty”[all fields] OR “school age”[all fields] OR “schoolage”[all fields] OR “school ages”[all fields] OR schoolage*[all fields] OR “one year old”[tw] OR “two year old”[tw] OR “three year old”[tw] OR “four year old”[tw] OR “five year old”[tw] OR “six year old”[tw] OR “seven year old”[tw] OR “eight year old”[tw] OR “nine year old”[tw] OR “ten year old”[tw] OR “eleven year old”[tw] OR “twelve year old”[tw] OR “thirteen year old”[tw] OR “fourteen year old”[tw] OR “fifteen year old”[tw] OR “sixteen year old”[tw] OR “seventeen year old”[tw] OR “eighteen year old”[tw] OR “1 year old”[tw] OR “2 year old”[tw] OR “3 year old”[tw] OR “4 year old”[tw] OR “5 year old”[tw] OR “6 year old”[tw] OR “7 year old”[tw] OR “8 year old”[tw] OR “9 year old”[tw] OR “10 year old”[tw] OR “11 year old”[tw] OR “12 year old”[tw] OR “13 year old”[tw] OR “14 year old”[tw] OR “15 year old”[tw] OR “16 year old”[tw] OR “17 year old”[tw] OR “18 year old”[tw] OR “two years old”[tw] OR “three years old”[tw] OR “four years old”[tw] OR “five years old”[tw] OR “six years old”[tw] OR “seven years old”[tw] OR “eight years old”[tw] OR “nine years old”[tw] OR “ten years old”[tw] OR “eleven years old”[tw] OR “twelve years old”[tw] OR “thirteen years old”[tw] OR “fourteen years old”[tw] OR “fifteen years old”[tw] OR “sixteen years old”[tw] OR “seventeen years old”[tw] OR “eighteen years old”[tw] OR “2 years old”[tw] OR “3 years old”[tw] OR “4 years old”[tw] OR “5 years old”[tw] OR “6 years old”[tw] OR “7 years old”[tw] OR “8 years old”[tw] OR “9 years old”[tw] OR “10 years old”[tw] OR “11 years old”[tw] OR “12 years old”[tw] OR “13 years old”[tw] OR “14 years old”[tw] OR “15 years old”[tw] OR “16 years old”[tw] OR “17 years old”[tw] OR “18 years old”[tw]))

## Appendix B. Risk of Bias of the Articles Included According to the ROBINS-I Criteria

**Article**	**Aydogmus et al.**	**Paci et al.**	**Wendling-Keim et al.**	**Balakumar et al.**
**Pre-intervention**				
Bias due to confounding	Moderate risk	Moderate risk	Moderate risk	Serious risk
Bias in selection of participants into the study	Low risk	Low risk	Low risk	Low risk
**At intervention**				
Bias in classification of interventions	Serious risk	Moderate risk	Serious risk	Serious risk
**Post-intervention**				
Bias due to deviations from intended interventions	Low risk	Low risk	Low risk	Low risk
Bias due to missing data	Low risk	Low risk	Low risk	Low risk
Bias in measurement of outcomes	Moderate risk	Moderate risk	Low risk	Moderate risk
Bias in selection of the reported result	Low risk	Low risk	Low risk	Low risk
**Total risk of bias score**	Serious risk	Moderate risk	Serious risk	Serious risk
